# Computer Vision for Predicting the Efficacy of Neoadjuvant Therapy in Breast Cancer

**DOI:** 10.3390/cancers18111857

**Published:** 2026-06-05

**Authors:** Daria Sitnikova, Alexey Fayzullin, Fedor Chistov, Peter Timashev, Nikita Savelov

**Affiliations:** 1 Institute for Regenerative Medicine, Sechenov University, 8-2 Trubetskaya St., 119991 Moscow, Russia; sitnikova_d_a1@student.sechenov.ru (D.S.); timashev_p_s@staff.sechenov.ru (P.T.); 2 Moscow City Oncology Hospital No. 62, 27 Istra, 143515 Moscow, Russia; chistovfd@zdrav.mos.ru (F.C.); savelovna1@zdrav.mos.ru (N.S.)

**Keywords:** breast cancer, neoadjuvant, pathological response, digital pathology, computer vision

## Abstract

Neoadjuvant therapy (NAT) is a standard component of breast cancer treatment, yet response rates vary substantially across patients. Accurate prediction of pathological complete response remains an unmet clinical need to improve patient selection for NAT. This review summarizes current approaches of using computer vision to predict breast cancer response to NAT from histopathological slides. We examined studies employing computer vision and machine learning models on hematoxylin and eosin and immunohistochemically stained whole-slide images, focusing on morphological features of tumor cells, stroma and tumor-infiltrating lymphocytes associated with pathological complete response. Key morphological predictors of therapy resistance included low tumor cell density with cord-like patterns, necrosis, predominance of collagenous and fibroblast-rich stroma and tumor vascularization, while therapy sensitivity was associated with high nuclear staining intensity, high tumor cell density and lymphocyte infiltration. We highlighted the advantages of incorporating multimodal data to enhance predictive performance. Our analysis demonstrates that computer vision models can detect subtle morphological patterns that may be difficult for pathologists to evaluate, providing valuable insights for personalized therapy planning in breast cancer. Further development of cross-modal, interpretable artificial intelligence solutions may improve prediction accuracy and deepen our understanding of tumor biology relevant to NAT response.

## Simple Summary

Breast cancer is the most common malignancy in women, and patients often receive neoadjuvant therapy (NAT) before surgery to reduce tumor burden and improve outcomes. However, not all patients respond to NAT, and its effectiveness remains difficult to predict. This review summarizes approaches using computer vision to predict breast cancer response to NAT from histopathological slides. The review is focused on biological insights revealed by computer vision and technical aspects of model development. Particular attention was given to tumor cells, stromal components and tumor-infiltrating lymphocytes, allowing a comprehensive analysis of the tumor and its microenvironment. Predictors of resistance included low tumor cell density with cord-like patterns, necrosis, vascularization and collagenous or fibroblast-rich stroma, whereas sensitivity was associated with dense tumor cell populations and lymphocyte infiltration. Our analysis demonstrates that computer vision can detect and measure subtle microstructural patterns and improve the prediction of NAT response.

## 1. Introduction

Breast cancer is the most common malignancy in women and the leading cause of cancer-related mortality worldwide [1]. Neoadjuvant therapy (NAT) for breast cancer is included in standard treatment protocols for patients with locally advanced disease and is administered prior to surgical resection of the tumor. NAT is indicated for the vast majority of patients with human epidermal growth factor receptor 2 (HER2)-positive and triple-negative breast cancer (TNBC), as well as for selected patients with estrogen receptor (ER)-positive/HER2-negative tumors who are at increased risk of metastatic relapse. However, the decision to prescribe NAT remains with the oncologist and depends on the characteristics and individual features of each patient [2].

NAT can reduce local tumor spread, thereby allowing for less extensive surgery. Several studies have even suggested the possibility of omitting surgery in patients who achieve an exceptional response to neoadjuvant chemotherapy [3]. NAT not only increases the rate of breast-conserving surgery and overall survival, but also reduces the risk of postoperative complications [4]. Nevertheless, NAT has certain limitations. Approximately 30% of patients do not respond to NAT, and only 10–50% of responders achieve a pathological complete response (pCR, defined as the absence of residual invasive cancer cells in both the breast and axillary lymph nodes) [5]. The ability to identify NAT sensitivity and predict the likelihood of achieving pCR at an early stage would enable more effective personalized treatment regimens, including timely therapy intensification for non-pCR patients [6].

Several clinical and biological markers correlate with tumor aggressiveness and, consequently, with patient sensitivity to neoadjuvant therapy (NAT) and are considered by oncologists when selecting a treatment strategy, including age, T stage, histological grade, pre-NAT Ki-67 index, and HER2 and ER status [7]. A especially important independent factor determining patient susceptibility to NAT is the tumor molecular subtype. According to one study [8], only 0.3% of patients with the luminal A subtype achieved pCR during NAT, whereas the corresponding rates for HER2-positive, TNBC, and luminal B subtypes were 40%, 23%, and 8%, respectively. However, likely due to the pronounced heterogeneity of breast cancer, therapeutic response remains difficult to predict.

In addition to the guidelines, several histopathological markers have been associated with response to therapy: lymphocyte infiltration of the tumor [9], tumor–stromal ratio [10] and the structural organization of the tumor tissue itself. The study of tumor morphology and the identification of relationships between specific histological markers and pCR represent an important and relevant problem in modern oncology.

Many researchers have attempted to incorporate artificial intelligence into algorithms for assessing the probability of pCR, primarily in breast cancer but also in colorectal [11], gastric [12], bladder [13] and several other cancers. The value of this approach lies in the ability of artificial intelligence to extract large amounts of data from whole-slide images (WSIs) and analyze them comprehensively, thereby identifying prognostically relevant patterns, some of which may be subtle and escape the attention of pathologists.

The aim of this review was to identify the predictively valuable histological features and effective approaches to the analysis of breast cancer and its microenvironment. We were interested not only in data on technical approaches to developing and training predictive models, but also in fundamental biological findings related to tumor sensitivity to NAT that computer vision may help to reveal. We structured our findings into three sections focusing on tumor cells (Table 1), extracellular matrix (Table 2) and lymphocyte infiltration (Table 3).

This review focused on studies investigating the application of computational pathology and computer vision approaches for predicting response to NAT in breast cancer patients. A literature search was conducted between 28 February 2026 and 1 April 2026 using the PubMed, Scopus and Web of Science databases. The search strategy included the following terms and their combinations: “digital pathology”, “computational pathology”, “computer vision”, “artificial intelligence”, “deep learning”, “breast cancer” and “neoadjuvant”. Additional relevant studies were identified through manual screening of reference lists from selected articles. Studies were considered eligible if they investigated computer vision approaches for predicting response to NAT using histopathological images of breast cancer. Original research articles focusing on breast cancer morphology, tumor microenvironment, lymphocyte infiltration or multimodal computational models were included. Studies focused exclusively on other tumor localizations, non-histopathological imaging modalities or unrelated clinical outcomes were excluded. Because of the rapidly evolving nature of computational pathology research, two studies identified during the literature search were available only as preprints at the time of manuscript preparation. These studies were included because of their direct relevance to the topic and their potential contribution to the ongoing scientific discussion; however, they had not yet undergone peer review and should therefore be interpreted with caution.

## 2. Prognostic Value of Tumor Cell Morphology

**Figure 1:**
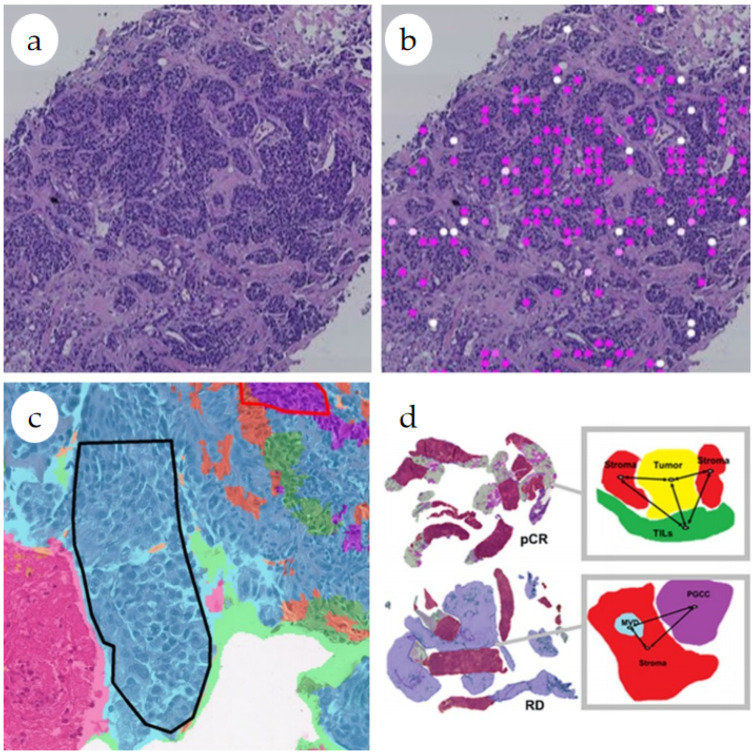
Examples of computer vision analysis of tumor tissue. (**a**) Example of tumor biopsy of a patient with pathological complete response (pCR). Adapted from [17] under the terms of the Creative Commons CC BY license https://creativecommons.org/licenses/by/4.0/. (**b**) Tumor epithelium map generated by a computer vision model, where pink and white dots represent high and low probabilities of high tumor aggressiveness and resistance to therapy, which correlate with probabilities of pCR or residual disease (RD), respectively. Adapted from [17] under the terms of the Creative Commons CC BY license https://creativecommons.org/licenses/by/4.0/. (**c**) Example of tumor tissue segmentation performed by a computational model trained on tissue annotations (drawn polygons: black: Tumor, red: Lymphocytes). Predicted tumor is hued blue. Adapted from [18] under the terms of the Creative Commons CC BY license https://creativecommons.org/licenses/by/4.0/. (**d**) Visualization of spatial feature analysis and graph construction performed by a computational model to assess how the proximity of histological classes correlates with tumor aggressiveness and prognosis. TILs—tumor-infiltrating lymphocytes; MVD—microvessel; PGCC—polyploid giant cancer cell. Adapted from [23] under the terms of the Creative Commons CC BY license https://creativecommons.org/licenses/by/4.0/.

Among the reviewed studies, we identified several main approaches to tumor tissue analysis for pCR prediction: (i) analysis of tumor nuclei and their characteristics; (ii) tumor tissue segmentation followed by feature extraction; and (iii) classification of slide fragments into multiple histological categories, with prognostically significant patterns identified specifically in tumor tissue. In some studies, histological image analysis was performed on pre-selected regions of interest (ROIs) representing tumor tissue, while in others the model was allowed to independently identify areas of interest and associate them with the probability of pCR. The morphological features generally considered when deciding on NAT include markers of tumor aggressiveness, such as degree of invasiveness, severity of cellular and nuclear polymorphism (Figure 1c) and mitotic activity. These parameters are assessed in accordance with the CAP protocol [14].

**Table 1:** Original studies on computer vision models for predicting pathological complete response (pCR) of breast cancer to neoadjuvant therapy focusing on tumor cell morphology.

References	Patients	Models	Data Modality	Training Data	Outcome Prediction
[15]	58 (38 HER2+, 20 TNBC)	(1) VGG19—tumor segmentation; (2) Three U-Net-based CNNs—nuclei segmentation; (3) Logistic regression—pCR/non-pCR classification at the patient level.	(1) H&E slides	(1) tiles with selected ROIs; (2) external data; (3) nuclear features; (4) pCR/non-pCR data.	Sensitivity = 79%
[16]	149	(1) U-Net—nuclear segmentation; (2) Gradient boosting machine (GBM) with decision trees—selection of significant variables and pCR/non-pCR classification at the patient level.	(1) H&E slides; (2) clinical data	(1) segmented tiles; (2) nuclear features; (3) clinicopathological data; (4) pCR/non-pCR data.	pCR; AUC = 0.90
[17]	540	Two Inception V3-based models. (1) CNN I—tumor tissue segmentation; (2) CNN II—pCR/non-pCR tile classification.	(1) H&E slides; (2) molecular subtype	(1) segmented tiles; (2) tumor molecular subtype; (3) sTIL density; (4) pCR/non-pCR data.	pCR; AUC = 0.89
[18]	721 (178 TNBC, 543 Luminal B)	(1) U-Net—segmentation of tumor, lymphocytes, stroma, necrosis, fat and other tissues; (2) External pre-trained model—mitosis detection; (3) Logistic regression—pCR/non-pCR classification at the patient level.	(1) H&E slides	(1) segmented WSIs; (2) 4 computed biomarkers; (3) pCR/non-pCR data.	pCR; AUC = 0.66–0.88, depending on biomarker and molecular subtype
[19]	126 (62 HER2+, 64 TNBC)	(1) DeepLabV3—segmentation and classification into stroma, tumor and lymphocyte aggregates; (2) K-means clustering—segmentation and classification into CD8, CD163 and PD-L1; (3) LASSO-regularized logistic regression—pCR/non-pCR classification at the patient level.	(1) H&E slides; (2) IHC slides; (3) clinical data (4) molecular subtype	(1) segmented H&E and IHC tiles with selected ROIs; (2) three categories of extracted features (area ratio, proportion, purity); (3) clinical data; (4) molecular subtype; (5) pCR/non-pCR data.	pCR; AUC = 0.90 (HER2+); AUC = 0.59 (TNBC)
[20]	76 (TNBC)	(1) MRCNN—detection of tumor cells, lymphocytes and IHC markers (Ki67+ and pH3+); (2) LDA, SVM, MLP—pCR/non-pCR classification at the patient level.	(1) H&E slides; (2) IHC slides	(1) triplets of tiles (H&E + IHC) with segmentation; (2) hotspot regions with the highest number of labeled markers; (3) pCR/non-pCR data.	pCR; AUC = 0.71
[21]	165 (TNBC)	(1) Deep CNN-Based model—tile-level pCR/non-pCR classification.	(1) H&E slides; (2) clinical data	(1) segmented tiles; (2) clinical data; (3) pCR/non-pCR data.	ypTNM stage classification; AUC = 0.88–0.73 for different stages
[22]	1670	(1) Nuclear-Segandcls model—segmentation and classification of epithelial, lymphocytes, tumor and no-label cells; (2) ResNet50—feature extraction; (3) Gaussian Context Transformer (GCT), CLAM—pCR/non-pCR classification at the patient level.	(1) H&E slides; (2) clinical data	(1) segmented tiles; (2) clinical data; (3) pCR/non-pCR data.	pCR; AUC = 0.821 (95% CI 0.763–0.878)
[23]	164 (TNBC)	(1) 1-nearest neighbor (1NN), linear support vector machine (linSVM), radial basis function SVM and ensemble tree (ensembleTree)—classification into 16 histological labels and classification of pCR/non-pCR at the patient level.	(1) H&E slides	(1) segmented tiles; (2) graphs; (3) pCR/non-pCR data.	pCR; AUC = 0.832 (95% CI 0.792–0.873)
[24]	874 (453 HR+ HER2−, 287 HER2+, 134 TNBC)	(1) ResNet18—classification into cancer cell predominant, stromal cell predominant and other; (2) Deep learning pathological model, Multivariate Logistic Regression Clinical Model—pCR/non-pCR classification at the patient level.	(1) H&E slides; (2) clinical data	(1) segmented tiles; (2) feature vectors; (3) clinical data; (4) TILs data; (5) pCR/non-pCR data.	pCR; AUC = 0.82 (95% CI 0.77–0.87)
[25]	310	(1) CNN—for classification into tumor/not tumor and into therapy into four response grades: RG0/1/2/3; (2) SVM and RF—classification into RG0/1/2/3.	(1) H&E slides; (2) molecular subtype	(1) WSI with selected ROIs; (2) segmented ROIs, (3) extracted nuclei features; (4) molecular subtype data; (5) RG0/1/2/3 data.	RG0/1/2/3 class assignment; CNN AUC = 0.9. SVM, RFM AUC = 0.949
[26]	596 (78 TNBC, 207 HER2+, 311 HR+/HER2−)	(1) CrossFormer + Squeeze and Excitation (SE) = SE-CrossT architecture model—feature extraction and pCR/non-pCR classification at the patient level.	(1) H&E slides; (2) ultrasound images; (3) clinical data	(1) tiles with selected ROIs; (2) ultrasound images; (3) clinical data; (4) pCR/non-pCR data.	pCR; AUC = 0.873 (95% CI 0.834–0.898)
[27]	440	(1) VGG16—feature extraction; (2) SVM classifier—pCR/non-pCR classification at the patient level.	(1) H&E slides; (2) clinical data; (3) molecular subtype	(1) tiles with selected ROIs (containing at least 50% of tumor tissue, necrosis and cell overlaps excluded); (2) clinical data; (3) molecular subtype data; (4) pCR/non-pCR data.	pCR; AUC = 0.76–0.78
[28]	201	(1) CellProfiler—feature extraction; (2) SVM—classification into 4 RCB groups (0–1—response to therapy, 2–3—resistance to therapy)	(1) H&E slides; (2) clinical data; (3) HER2+ status	(1) screenshots with the highest number of tumor cells; (2) clinical data; (3) HER2+ status; (4) RCB data.	RCB 0–I class assignment; AUC = 0.8301 (95% CI 0.7219–0.9382)
[6]	928	First approach: (1) EfficientNetB7—tiling and feature extraction; (2) Multilayer Perceptron (MLP)—tile classification into 3 groups pCR/RD (Residual Disease); (2) SoftMax layer—pCR/RD classification at the patient level. Second approach: (1) Vision Transformer (ViT)—tiling and feature extraction; (2) MLP—pCR and RD classification	(1) H&E slides; (2) clinical data; (3) molecular subtype	(1) unlabeled WSI; (2) clinical data; (3) molecular subtype data; (4) pCR/non-pCR data.	pCR; AUC = 0.59–0.87 depending on molecular subtype

Analysis focused exclusively on tumor nuclei was performed in two studies by Saednia K., Dodington DW. and Lagree A. [15,16]. In these works, the researchers extracted features after nuclear segmentation and then identified the most prognostically relevant features using gradient boosting and logistic regression. They demonstrated that high mean nuclear intensity and low heterogeneity of nuclear texture (GLCM-COR feature) were associated with pCR, and also identified an association of pCR with nuclear density and nuclear orderliness. This approach can be attributed to explanatory artificial intelligence, since morphological criteria were established mathematically rather than described by pathologists using models such as GradCAM. In a subsequent study [16], the model was developed using a substantially larger set of nuclear features and achieved high predictive performance (area under the curve, AUC = 0.90) in a limited patient cohort.

To capture not only the morphology of individual tumor cells but also the architecture of the entire tumor tissue, features can be extracted from small patches of WSIs, referred to as tiles, rather than from individual cells. In the study by Li F. and Yang Y. [17], the model was trained exclusively on segmented tumor tissue and information regarding the presence or absence of pCR. The model first predicted therapy response at the tile level and subsequently at the patient level, deriving a so-called pCR score, which represented the average probability of response across all tiles from a single patient (Figure 1a,b). After integrating the extracted tumor features with clinical data and molecular subtype information, the model achieved an area under the curve (AUC) of 0.89. This approach demonstrates that tumor cell morphology alone contains information with independent prognostic value. However, the study did not provide an interpretation of the identified morphological features.

At the same time, most studies on this topic employed multi-class labeling. Typically, to predict pCR, they included segmentation of tumor tissue, stroma and tumor-infiltrating lymphocytes (TILs), and used convolutional neural networks (CNNs) for feature extraction and classification. In the study by Aswolinskiy W. and Munari E. [18], features were extracted from segmented tissue to generate four numerical biomarkers, three characterizing lymphocytes and one representing tumor mitoses (Figure 1d). Although all biomarkers were statistically significant predictors (*p* < 0.05) with AUC values above 0.5, their overall predictive ability was low. The prognostic value of mitotic activity did not show clear prognostic value in any patient subgroup.

A different approach to multi-class labeling was taken by Huang Z. and Shao W. [19]. In the study, the researchers also used CNNs for extraction and classification of tumor cell features, but they trained the model on immunohistochemically (IHC) stained slides of breast cancer. The authors quantified the expression of PD-L1 and several other markers according to specific criteria: area ratio (reflecting local expression saturation), proportion (indicating predominant sites of expression) and purity (indicating marker content relative to others in the region), as well as its localization across histological classes: tumor, stroma and lymphocytes. As expected, increased PD-L1 content in tumor tissue was associated with a higher probability of therapy resistance. Notably, PD-L1 had the greatest prognostic significance in TNBC: favorable markers included PD-L1 ratio and proportion in lymphocytes, whereas the unfavorable marker was PD-L1 ratio in tumor tissue. For other molecular subtypes of breast cancer, however, immune cell markers were more prognostically relevant. Another study that combined hematoxylin and eosin (H&E) and IHC slide data was conducted by Bhattarai S. and Saini G. [20] (currently preprint). In this case, the IHC markers reflected proliferative activity of tumor tissue: Ki67+ and pH3+. These markers, however, did not demonstrate prognostic significance. The best performance was achieved by a model trained on features of TILs (AUC = 0.71), while the inclusion of IHC data reduced the predictive potential of the model.

Other multi-label studies on breast cancer morphology combine detailed annotation with tile-level analysis. In the study by Krishnamurthy S. and Jain P. [21], a CNN was trained on labeled images with segmentation of tumor, necrosis, stroma, lymphocytes, adipose tissue and healthy glandular tissue. The model analyzed segmented tiles, classified them into groups by morphological similarity (based on the predominant class of the object), and then by pCR/non-pCR status to estimate the probability of complete response at the tile level. Classifying tiles by pCR score allowed pathologists to identify features the model focused on during tissue analysis: a low level of necrosis was noted in the pCR group. The study also showed that predictive accuracy varied according to tumor stage, with AUC values ranging from 0.88 to 0.73.

A CNN trained on tiles with multi-class labeling of not just different histological classes, but individual cells—epithelial, tumor and lymphocytes, was described in the article by Yang P. and Mao N. [22]. Therapy response prediction was performed using CLAM (a type of multiple instance learning). After combining the extracted cellular composition features with clinical data and selecting the most relevant variables, the model achieved an AUC of 0.821. Retrospective heat map analysis revealed that the pCR group more often included tiles in which lymphocytes quantitatively predominated over tumor cells, whereas tiles with a predominance of tumor cells were largely attributed to the therapy-resistant group.

Researchers Fisher T.B. and Saini G. [23] took multi-class labeling further and employed a large-scale labeling protocol, incorporating as many as 16 histological classes. Instead of CNNs, however, four machine learning classifiers were tested, with the radial basis function support vector machine (SVM) achieving the best performance for predicting therapy response (AUC = 0.832). A distinctive feature of this work was the use of graph construction to derive pairs of histological classes whose spatial proximity was most prognostically relevant. It was shown that tumors infiltrated with lymphocytes were associated with pCR, whereas extensive tumor vascularization was associated with resistance to therapy.

An effective strategy for integrating annotation with tile-level analysis is the generation of heat maps that visualize the predominant class distribution within each tile. In the article by Li B. and Li F. [24], labeling also included multiple histological classes, and the goal was to classify pre-segmented tissue tiles by their predominant tissue component: cancer cell predominant, stromal cell predominant or other. Subsequent feature selection and pCR/non-pCR classification were performed using logistic regression models. Interpretation of the results revealed that the model focused primarily on vacuolated areas around tumor cells, mitotic figures and pleomorphic nuclei with multiple atypical nucleoli. However, the authors did not examine how these morphological markers specifically related to therapy response. In addition to morphological features, the model incorporated clinical data and pathologist-assigned TILs scores, achieving an AUC of 0.82.

In a number of studies, models were trained not on fully segmented tissue but on pre-selected ROIs that most comprehensively reflected tumor morphology. Typically, pathologists identified areas with high cellularity, containing at least 50% tumor tissue. In the study by Saednia K. and Lagree A. [15], a CNN was applied to such selected tiles (>50% tumor tissue, <10% background, including tumor edges) to extract features. In the article by Shen B. and Saito A. [25], the authors emphasized parameters of tissue and nuclear atypia and designed a multi-stage pipeline in which ROIs were passed sequentially through CNN, SVM and random forest (RF) models. They argued that the relatively low accuracy of prior studies was due to the complexity of tumor atypia and heterogeneity. Their solution was to assign CNN the task of capturing structural atypia patterns, while SVM and RF were used to analyze nuclear atypia. In the first stage, CNN classified unlabeled ROIs as tumor or non-tumor and further stratified them by probability into four response grades (RG0-3). In the next two stages, the model processed labeled ROIs and pre-extracted nuclear atypia parameters, while also incorporating molecular subtype data. This approach achieved an AUC of 0.949. However, it should be noted that the study did not use the standard binary classification of pCR/non-pCR; instead, it stratified patients into four response grades based on the Japanese Breast Cancer Society criteria.

A key limitation of diagnostic breast cancer biopsies is that, as thin tissue samples, they cannot fully capture the tumor’s microstructure; therefore, combining them with volumetric data can substantially enhance the accuracy of NAT prediction. The article by Guo J. and Chen B. [26] incorporated both tiles with selected ROIs as well as ultrasound images of the tumor and adjacent tissue in their model’s training. Heat map analysis revealed that tiles with pronounced invasive carcinoma were most prognostically informative, while in ultrasound, the most predictive regions were tumor boundaries and internal greyscale structures. This cross-modal approach, combined with clinical data, yielded strong predictive performance (AUC = 0.873).

Zeng H. and Qiu S. [27] trained their model on ROI-selected tiles (containing ≥50% tumor tissue, excluding necrosis and overlapping cells). The model independently identified tile-level patterns associated with pCR, though the authors did not analyze feature-level interpretability. By combining ROI-derived features with clinical and molecular subtype data, their SVM classifier achieved an AUC of 0.76–0.78 for test groups. A related approach was described by Li H. and Ju X. [28], who trained their model on ROI-based screenshots and used SVM for response prediction. Here, classification was performed into four groups according to residual cancer burden (RCB): RCB 0–1 (response to therapy) vs. RCB 2–3 (resistance). From numerous extracted features, nine were selected as most prognostically relevant, largely reflecting tissue texture and “granularity.” Although the authors did not establish direct links between these morphological markers and NAT response, their results suggest that tumor tissue contains subtle, non-intuitive patterns—imperceptible to the human eye—that may indicate the likelihood of achieving pCR.

Finally, in the preprint by Valderrama N.F. and Morel L. [6], the authors abandoned both WSI segmentation and ROI selection, instead allowing the EfficientNet computer vision model to autonomously learn and identify patterns within tiles. The model’s predictive performance varied by molecular subtype (AUC = 0.59–0.87), with the best results observed in ER+/HER2− tumors and the poorest in TNBC. Retrospective analysis demonstrated that morphological predictors of pCR differ by molecular subtype. Features such as large nuclei with heterogeneous chromatin, high cellular density, multiple mitoses and pronounced inflammatory infiltration were primarily associated with pCR, whereas cord-like structures, small nuclei with homogeneous chromatin, low cellular density and a prominent stromal component were associated with resistance. In TNBC specifically, high cell density and marked structural pleomorphism were identified as indicators of therapy sensitivity.

Another rationale for analyzing breast cancer at the level of individual tumor cells is the marked reduction in prognostic value observed in low-resolution images. Li F. and Yang Y. [17] reported that high predictive efficiency was achieved only when the model was trained on a sufficiently large number of tiles (≥500). Zeng H. and Qiu S. [27] found that predictive accuracy was highest when training on tiles at ×20 magnification (among ×4, ×10, ×20 and ×40). This may reflect an optimal balance between resolution and the amount of tissue captured per ROI. At ×4 and ×10 magnifications, although images contained sufficient tumor tissue, the resolution was insufficient for extracting detailed morphological features. At ×40 magnification, the model captured fine cellular details but included too little tissue to account for broader structural and organizational features of the tumor.

In conclusion, several morphological patterns are consistently associated with resistance to NAT: heterogeneous nuclear texture, low tumor cell density and cord-like architecture, extensive necrosis, pronounced vascularization and tumor-specific PD-L1 expression. Conversely, intense nuclear staining and high levels of lymphocyte infiltration of the tumor are associated with pCR. Furthermore, genetic analysis [22] revealed that patients achieving pCR exhibited higher expression of genes linked to immune response potentiation. It is also noteworthy that most studies incorporated multimodal data, combining morphological features with clinical information, molecular subtype or pathologist-assessed lymphocyte scores. Among the three studies in which models were trained exclusively on labeled tiles with pCR/non-pCR outcomes, the highest reported AUC was 0.827 [23]. Finally, predictive accuracy has been shown to depend on breast cancer subtype. TNBC remains the most challenging to predict, likely due to its pronounced intratumoral heterogeneity.

## 3. Prognostic Value of Extracellular Matrix Morphology

Stroma is not as commonly investigated for predicting response to NAT as tumor tissue or TILs. Moreover, international protocols guiding tumor assessment and NAT selection for breast cancer patients do not include stromal parameters. Nevertheless, specific stromal characteristics may provide insights into the tumor’s molecular subtype: lymphocyte-rich stroma is typical of HER2+ and TNBC, whereas a predominance of collagen and fibroblasts is more characteristic of ER+ subtypes [29]. The amount of stroma may also have prognostic value. For instance, one study [30] investigated the relationship between the stroma–tumor ratio (STR, the proportion of stroma to tumor cells) and patient prognosis across molecular subtypes. A high STR was associated with a favorable prognosis in luminal A cancers, with an unfavorable prognosis in TNBC and showed no prognostic impact in HER2+ tumors. Therefore, the type, quantity and structural features of the stromal component cannot be regarded as insignificant, and their association with NAT sensitivity warrants further investigation.

**Table 2:** Original studies on computer vision models for predicting pathological complete response (pCR) of breast cancer to neoadjuvant therapy focusing on extracellular matrix.

References	Patients	Models	Data Modality	Training Data	Outcome Prediction
[31]	1035	(1) E-S classifier—stroma segmentation; (2) Inception-V4—pCR/non-pCR classification at the tile level; (3) Logistic regression—pCR/non-pCR classification at the patient level.	(1) H&E slides; (2) clinical data	(1) tiles with selected ROIs to predict; (2) clinicopathological data; (3) TILs scores; (4) pCR/non-pCR data.	pCR; AUC = 0.788 (95% CI 0.783–0.793)
[32]	120 (TNBC)	(1) Random Forest (RF)—pixel-level classification into myxoid stroma, collagenous stroma, Tumor, immune stroma; (2) LASSO regression—classification into significant and insignificant by association with pCR; (3) Decision Tree Classifier—classification of pCR/non-pCR at the patient level	(1) H&E slides; (2) clinical data	(1) segmented tiles; (2) clinicopathological data; (3) pCR/non-pCR data.	pCR; AUC = 0.74–0.78. Poor outcomes; AUC = 0.62
[21]	165 (TNBC)	Deep CNN-Based model—tile-level pCR/non-pCR classification.	(1) H&E slides; (2) clinical data	(1) segmented tiles; (2) clinical data; (3) pCR/non-pCR data.	ypTNM stage classification; AUC = 0.88–0.73 for different stages
[6]	928	First approach: (1) EfficientNetB7—tiling and feature extraction; (2) Multilayer Perceptron (MLP)—tile classification into 3 groups pCR/RD (Residual Disease); (2) SoftMax layer—pCR/RD classification at the patient level. Second approach: (1) Vision Transformer (ViT)—tiling and feature extraction; (2) MLP—pCR and RD classification	(1) H&E slides; (2) clinical data; (3) molecular subtype	(1) unlabeled WSI; (2) clinical data; (3) molecular subtype data; (4) pCR/non-pCR data.	pCR; AUC = 0.59–0.87 depending on molecular subtype

**Figure 2:**
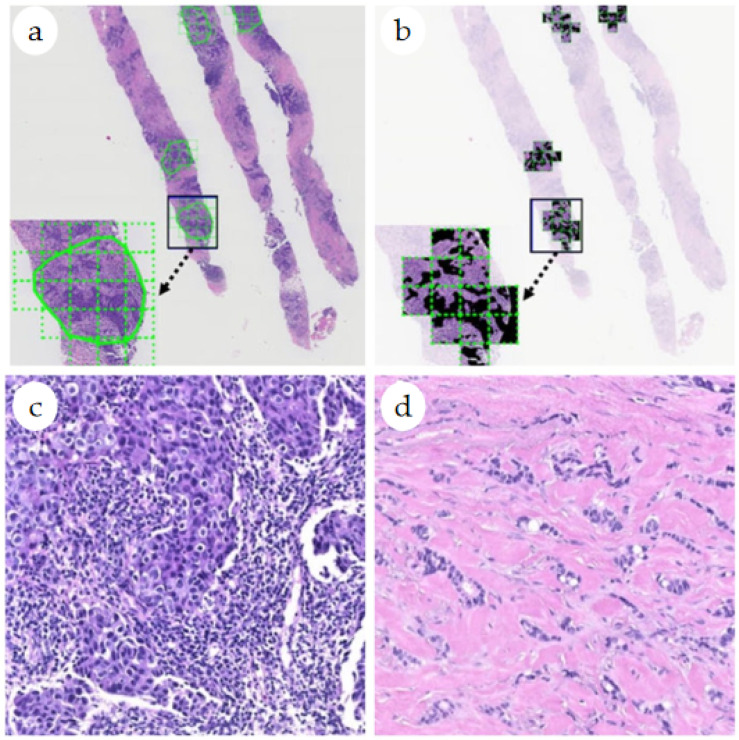
Computer vision analysis of extracellular matrix features associated with breast cancer response to neoadjuvant therapy. (**a**) Manually annotated stromal regions of interest. Adapted from [31] under the terms of the Creative Commons CC BY license https://creativecommons.org/licenses/by/4.0/. (**b**) Stromal tiles identified and selected by the computer vision model. Adapted from [31] under the terms of the Creative Commons CC BY license https://creativecommons.org/licenses/by/4.0/. (**c**) Lymphocyte-dominant stroma (L type). Adapted from [31] under the terms of the Creative Commons CC BY license https://creativecommons.org/licenses/by/4.0/. (**d**) Collagen-dominant stroma (C type). Adapted from [31] under the terms of the Creative Commons CC BY license https://creativecommons.org/licenses/by/4.0/.

Two studies specifically examined the stromal component of tumor biopsies. In the article by Li F. and Yang Y. [31], the authors applied two CNNs to segment tumor stroma (Figure 2a,b), after which they derived a tumor-associated stroma score—a metric of extracellular matrix characteristics reflecting the probability of pCR at both the tile and patient levels. When combined with clinical and pathological data, the model achieved an AUC of 0.788 for validation cohorts. Retrospective analysis further revealed biologically interpretable associations: stroma enriched with lymphocytes correlated with pCR (Figure 2c), whereas a collagen-dominant stroma was linked to resistance (Figure 2d). A strong emphasis on the stromal component was also made by Hacking S.M. and Karam J. [32], who trained a RF model for pixel-level classification of scanned images into four categories: myxoid stroma, collagenous stroma, tumor and immune stroma. Using statistically selected features together with clinicopathological data, a decision tree classifier was applied for pCR versus non-pCR prediction. When all features were considered, the model achieved a relatively low AUC of 0.62. However, when the prediction was based solely on the percentage of collagenous stroma per slide, the AUC increased to 0.78. Moreover, the study demonstrated that predominance of immune stroma and low levels of collagenous and myxoid components were associated with a higher probability of pCR and more favorable prognosis.

In other studies, the stromal component represented only one aspect of the broader morphological landscape considered for prognosis. In the previously discussed work of Krishnamurthy S. and Jain P. [21], stroma was included in a labeling protocol comprising seven histological classes. The authors described a highly automated model capable of classifying tiles based on morphological similarity, extracting feature vectors and predicting pCR at the tile level, while also integrating these features with clinical data to predict response at the patient level. However, the model architecture was not reported in sufficient detail. Tile classification by probability of response highlighted relevant morphological patterns, revealing an association between pCR and low levels of stromal fibrosis.

In the previously mentioned article by Valderrama N.F. and Morel L. [6], tiles across the entire whole-slide image, including surrounding tissue, were analyzed without segmentation or ROI selection. Distinct morphological patterns were decisive for prognosis depending on the molecular subtype. Inflamed, lymphocyte-infiltrated stroma emerged as an important predictor of pCR, particularly in HER2+ and TNBC. In contrast, fibrous and collagenous stroma was consistently associated with resistance to therapy across all molecular subtypes.

Taken together, these findings suggest that pronounced collagenous and fibrous stroma in tumor biopsies is indicative of a higher likelihood of resistance to NAT. In nearly all studies, models were trained on multimodal data, which improved predictive accuracy. However, this also underscores that morphological characteristics alone are insufficient for precise prediction of therapy response. Nevertheless, features embedded in the tumor microenvironment may serve as valuable complementary markers of pCR, enhancing the comprehensiveness of predictive models.

## 4. Prognostic Value of Lymphocyte Localization and Density

Lymphocyte infiltration is an important predictor of complete response to NAT. The term tumor-infiltrating lymphocytes (TILs) refers to lymphocytes observed in tumor biopsies, which some researchers further classify into sTILs and tTILs, denoting stromal and intratumoral infiltration, respectively. Quantifying TILs is a key step in the pathological assessment of tumor biopsies and plays a role in decisions regarding NAT prescription [2]. Both intratumoral and stromal lymphocyte infiltration are recognized as favorable histopathological features associated with less aggressive, therapy-responsive tumors and a higher probability of pCR [33]. However, several studies suggest that the predictive value of TILs varies by molecular subtype: they are strong predictors of pCR in HER2+ and TNBC tumors, but not in ER+ cancers [34]. Notably, in ER+ subtypes, pronounced lymphocyte infiltration is generally uncommon [35]. In addition, one study [36] demonstrated through multivariate analysis that the CD8+ TIL subset was an independent predictor of response to therapy, in contrast to CD4+ and FOXP3+ subsets.

In many respects, lymphocyte infiltration is the most apparent and visually recognizable morphological feature indicative of potential tumor sensitivity to therapy. Nevertheless, quantification of lymphocytes is often challenging for pathologists, both due to the difficulty of consistently assessing highly repetitive elements and the ambiguity of the TILs definition itself. Incorporating computer vision into the assessment of lymphocyte infiltration may help overcome these challenges and significantly improve the accuracy of pCR prediction.

**Table 3:** Original studies on computer vision models for predicting pathological complete response (pCR) of breast cancer to neoadjuvant therapy focusing on tumor-infiltrating lymphocytes.

References	Patients	Models	Data Modality	Data	Outcome Prediction
[24]	874 (453 HR+ HER2−, 287 HER2+, 134 TNBC	(1) ResNet18—classification into cancer cell predominant, stromal cell predominant and other; (2) Deep learning pathological model, Multivariate Logistic Regression Clinical Model—pCR/non-pCR classification at the patient level.	(1) H&E slides; (2) clinical data	(1) segmented tiles; (2) feature vectors; (3) clinical data; (4) TILs data; (5) pCR/non-pCR data.	pCR; AUC = 0.82 (95% CI 0.77–0.87)
[26]	596 (78 TNBC, 207 HER2+, 311 HR+/HER2−)	(1) CrossFormer + Squeeze and Excitation (SE) = SE-CrossT architecture model—feature extraction and pCR/non-pCR classification at the patient level.	(1) H&E slides; (2) ultrasound images; (3) clinical data	(1) tiles with selected ROIs; (2) ultrasound images; (3) clinical data; (4) pCR/non-pCR data.	pCR; AUC = 0.873 (95% CI 0.834–0.898)
[37]	113	(1) CNN11—classification into tumor cells, lymphocytes, stromal cells and other cells; (2) Logistic regression—pCR/non-pCR classification at the patient level.	(1) H&E slides	(1) WSIs with selected ROIs; (2) pCR/non-pCR data.	pCR; AUC = 0.709 (95% CI 0.659–0.879)
[38]	402	(1) ResNet-34—detection of tumor cells and lymphocytes; (2) DeepLabv3 and EfficientNet-B3—classification of pixels into cancer area, cancer stroma or other.	(1) H&E slides	(1) segmented tiles; (2) sTIL score; (3) pCR/non-pCR data.	-
[21]	165 (TNBC)	(1) Deep CNN-Based model—tile-level pCR/non-pCR classification.	(1) H&E slides; (2) clinical data	(1) segmented tiles; (2) clinical data; (3) pCR/non-pCR data.	ypTNM stage classification; AUC = 0.88–0.73 for different stages
[18]	721 (178 TNBC, 543 Luminal B)	(1) U-Net—segmentation of tumor, lymphocytes, stroma, necrosis, fat and other tissues; (2) External pre-trained model—mitosis detection; (3) Logistic regression—pCR/non-pCR classification at the patient level.	(1) H&E slides	(1) segmented WSIs; (2) 4 computed biomarkers; (3) pCR/non-pCR data.	pCR; AUC = 0.66–0.88, depending on biomarker and molecular subtype
[39]	765	(1) SVM—for classification into cancer, stromal or lymphocyte.	(1) H&E slides	(1) segmented tiles.	-
[23]	164 (TNBC)	(1) 1-nearest neighbor (1NN), linear support vector machine (linSVM), radial basis function SVM and ensemble tree (ensembleTree) with the RUSBoost method—classification into 16 histological labels and classification of pCR/non-pCR at the patient level.	(1) H&E slides	(1) segmented tiles; (2) graphs; (3) pCR/non-pCR data.	pCR; AUC = 0.832 (95% CI 0.792–0.873)
[19]	126 (62 HER2+, 64 TNBC)	(1) DeepLabV3—segmentation and classification into stroma, tumor and lymphocyte aggregates; (2) K-means clustering—segmentation and classification into CD8, CD163 and PD-L1; (3) LASSO-regularized logistic regression—pCR/non-pCR classification at the patient level.	(1) H&E slides; (2) IHC slides; (3) clinical data; (4) molecular subtype	(1) segmented H&E and IHC tiles with selected ROIs; (2) three categories of extracted features (area ratio, proportion, purity); (3) clinical data; (4) molecular subtype; (5) pCR/non-pCR data.	pCR; AUC = 0.90 (HER2+); AUC = 0.59 (TNBC)
[20]	76 (TNBC)	(1) MRCNN—detection of tumor cells, lymphocytes and IHC markers (Ki67+ and pH3+); (2) LDA, SVM, MLP—pCR/non-pCR classification at the patient level.	(1) H&E slides; (2) IHC slides	(1) triplets of tiles (H&E + IHC) with segmentation; (2) hotspot regions with the highest number of labeled markers; (3) pCR/non-pCR data.	pCR; AUC = 0.71
[22]	1670	(1) Nuclear-Segandcls model—segmentation and classification of epithelial, lymphocytes, tumor and no-label cells; (2) ResNet50—feature extraction; (3) Gaussian Context Transformer (GCT), CLAM—pCR/non-pCR classification at the patient level.	(1) H&E slides; (2) clinical data	(1) segmented tiles; (2) clinical data; (3) pCR/non-pCR data.	pCR; AUC = 0.821 (95% CI 0.763–0.878)

The importance of intratumoral lymphocytes as a predictive marker is underscored by the fact that even in studies where the authors did not explicitly aim to analyze lymphocyte infiltration, it nonetheless emerged as a characteristic associated with therapy-sensitive patients. For example, in the study by Li B. and Li F. [24], previously discussed in the section on tumor morphology, the model classified tiles according to the relative predominance of tumor and stroma. However, the interpretation of the model’s output revealed that it paid the greatest attention to regions with lymphocyte infiltration and aggregation. Moreover, the lymphocyte score independently calculated by pathologists was incorporated into the final prediction of therapy response, together with morphological and clinical features, yielding an AUC of 0.82. Similarly, in another already mentioned article by Guo J. and Chen B. [26], the primary focus was not on lymphocytes: ROIs were selected for training based on the most prominent invasive tumor areas. Yet, subsequent performance analysis demonstrated that ROIs enriched with TILs were the most prognostically informative. This study also integrated ultrasound examination results and clinical data, and this cross-modal approach achieved relatively high predictive accuracy (AUC = 0.873).

**Figure 3:**
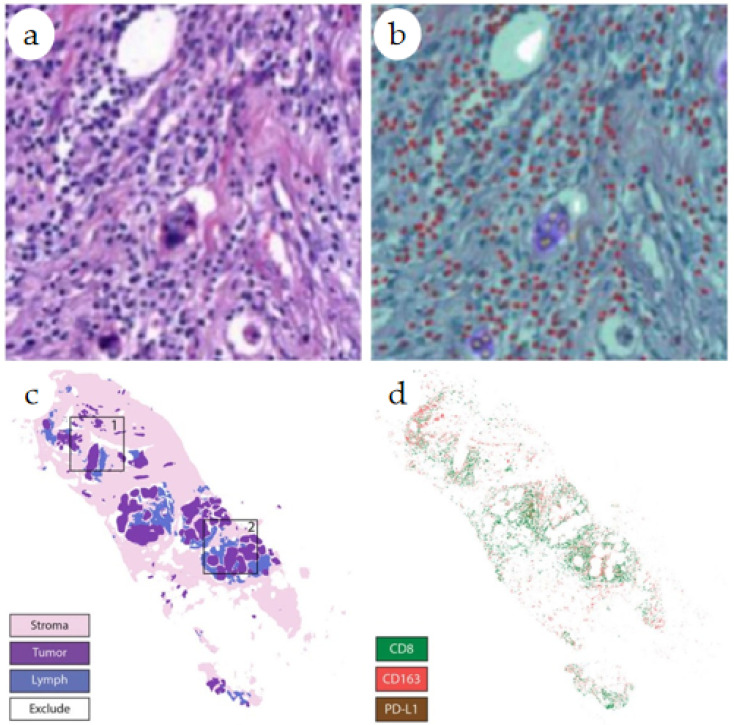
Computer vision analysis of immune cell features associated with breast cancer response to neoadjuvant therapy. (**a**) Region representing high tumor-infiltrating lymphocytes density. Adapted from [38] under the terms of the Creative Commons CC BY license https://creativecommons.org/licenses/by/4.0/. (**b**) Detected tumor-infiltrating lymphocytes (red). Adapted from [38] under the terms of the Creative Commons CC BY license https://creativecommons.org/licenses/by/4.0/. (**c**) Hematoxylin and eosin-stained tissue with segmentation masks of stroma, tumor and lymphocyte aggregates. Adapted from [19] under the terms of the Creative Commons CC BY license https://creativecommons.org/licenses/by/4.0/. (**d**) Immunohistochemically stained tissue with segmentation masks of CD8-, CD163- and PD-L1-positive cells. Adapted from [19] under the terms of the Creative Commons CC BY license https://creativecommons.org/licenses/by/4.0/.

In contrast, some studies focused exclusively on TILs, primarily to compare the accuracy of TIL assessment by pathologists with that of computational methods. Fanucci K.A. and Bai Y. [37] introduced the metric easTILs%, defined as the percentage of stromal area occupied by lymphocytes. The authors found that easTILs% calculated by a CNN achieved higher predictive accuracy (AUC = 0.709) compared to sTILs% assessed by pathologists (AUC = 0.627). This highlights the potential advantages of computational models in detecting subtle morphological patterns and extracting quantitative features in tasks involving repetitive histological elements. In the work by Choi S. and Cho S.I. [38], both pathologist-based and AI-assisted TIL assessments were compared (Figure 3a,b). In this case, however, the two approaches were used complementarily: when discrepancies occurred between the model and pathologist evaluations, the slides were re-reviewed and the lymphocyte score was adjusted. This strategy not only improved pathologist efficiency but also highlighted the limitations of deep learning models—such as missing lymphocytes and tumor cells or misclassifying atypical cells as lymphocytes. The study further confirmed the association of sTIL-high (sTIL ≥ 50) with a better response to NAT, although it did not specifically aim to predict pCR.

Other studies analyzed several histological classes of tissues and cells, including TILs. These articles differed in their approaches to feature extraction and evaluation, as well as in the datasets used to train their models. The previously mentioned study by Krishnamurthy S. and Jain P. [21] described a highly automated model capable of classifying tiles into groups based on morphological similarity. Classifying tiles according to the probability of response to therapy enabled pathologists to focus on specific morphological patterns. Tumor fragments with a high number of TILs were classified by the model as potentially sensitive to therapy, whereas tumor fragments without TILs or with only low levels of them, were classified as chemoresistant.

A CNN trained on labeled slides was also employed in the study by Aswolinskiy W. and Munari E. [18]. In this case, however, the researchers did not simply extract features from segmented tissue; instead, they derived three numerical biomarkers from segmentation maps, characterizing the degree of lymphocyte infiltration and the “inflammation” of tumor tissue. Logistic regression analysis showed that all three biomarkers were statistically significant predictors (*p* < 0.05) with AUC values above 0.5, although their predictive power remained limited. By comparison, simple visual assessment of lymphocytes on slides by pathologists demonstrated greater prognostic accuracy. Nevertheless, among the studied markers, those reflecting the number of TILs and the ratio of inflamed to non-inflamed tumor tissue proved most relevant for prognosis.

Multi-class labeling was also performed in the study by Ali H.R. and Dariush A. [39]. However, in this case the model based on SVM evaluated not only the abundance but also the localization of classes. The researchers found that higher lymphocyte density on a slide was associated with a greater likelihood of achieving pCR. Moreover, in an analysis of 557 post-therapy samples, they observed that a decrease in lymphocyte density after treatment was associated with complete response to NAT. Spatial relationships between histological classes were explored in greater detail in the previously discussed article by Fisher T.B. and Saini G. [23], which employed a comprehensive labeling protocol and graph-based analysis. Their findings indicated that pCR was associated with lymphocyte-infiltrated tumor and stroma, as well as with the spatial proximity of polyploid giant tumor cells and intratumoral lymphocytes. Conversely, proximity of stromal TILs to adipocytes was identified as a marker of resistance. In this study, four machine learning classifiers were tested, with the radial basis function SVM achieving the highest predictive accuracy (AUC = 0.832).

Authors Huang Z. and Shao W. [19] incorporated IHC slides in their research, with not only the PD-L1 expression in tumor tissue, but also CD8 (a cytotoxic T-cell marker), as well as CD163 being included in the segmentation protocol. Using CNN and K-means clustering, both H&E and IHC slides were successfully segmented, which improved the accuracy of conclusions regarding the morphological and molecular composition of the tissue (Figure 3c,d). Tissue was segmented into three categories: tumor, stroma and lymphocytes aggregated regions. High expression of cytotoxic T-cell and macrophage markers within lymphocyte aggregates was associated with pCR, whereas a high proportion of cytotoxic T-cells in stroma, compared with other regions, was linked to resistance. The study also demonstrated that the prognostic significance of markers varied by molecular subtype: the resulting model demonstrated high accuracy for HER2+ tumors (AUC = 0.90) but substantially lower accuracy for TNBC (AUC = 0.59).

The study by Bhattarai S. and Saini G. [20] was notable for defining tumor and stromal lymphocytes (tTILs and sTILs) as two distinct groups, based on whether lymphocytes were in contact with the tumor. Features characterizing lymphocyte infiltration, as well as other morphological features, were extracted from H&E WSIs. As a result, the model trained solely on the tTIL feature set achieved the best performance (AUC = 0.71), followed by the combined sTIL + tTIL model (AUC = 0.59).

In the article by Yang P. and Mao N. [22], as it was already mentioned, a CNN was trained on tiles with multi-class annotation of individual cells (epithelial, tumor and lymphocytes). After combining the extracted cellular composition features with clinical data and selecting the most informative variables, the researchers achieved an AUC of 0.821. Beyond model training, genetic and IHC analyses were performed on 51 patients with high or low pCR probabilities. The study found that the CXCL10 and CD2 genes, which encode inflammatory mediators, were associated with therapy sensitivity. The IHC analysis revealed that tumors sensitive to therapy typically contained abundant plasma cells and activated CD4 lymphocytes, while a high density of macrophages within the tumor was associated with resistance.

Taken together, these findings indicate that lymphocyte infiltration of both tumor and stroma may be a favorable prognostic feature associated with higher pCR probability. However, some evidence suggests that expression of cytotoxic T-cell markers in stroma could possibly be linked to poor outcomes and resistance, underscoring the importance of assessing not only the intensity and localization of the immune response but also the composition of lymphocyte infiltrates. This group of studies includes the largest number of projects in which models were trained exclusively on WSI data, suggesting that TILs may function as a more independent prognostic feature compared with other histological characteristics.

## 5. Discussion

**Figure 4:**
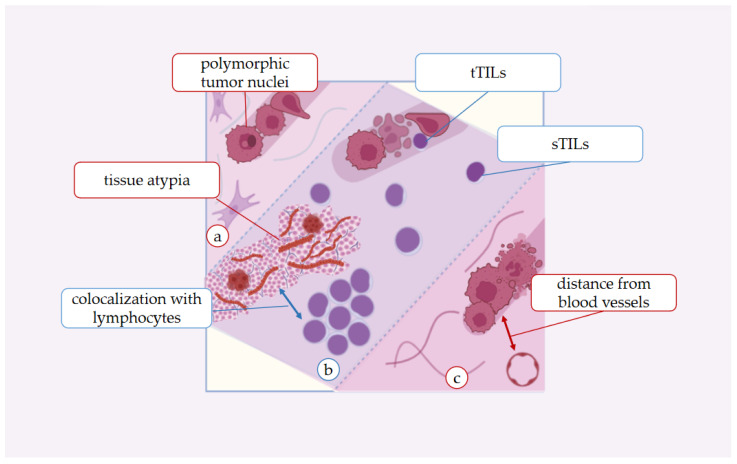
Cell- and tissue-level features in histological slides breast cancer analyzed by computer vision. (a) Fibroblast-dominant stroma (F type). (b) Lymphocyte-dominant stroma (L type). (c) Collagen-dominant stroma (C type). Blue and red represent features associated with high tumor aggressiveness and resistance to therapy, respectively. tTILs—tumor-infiltrating lymphocytes within tumor region. sTILs—tumor-infiltrating lymphocytes within stromal region. Created with BioRender.com.

One of the key conclusions of this review is that model training should account not only for the quantity of tumor tissue present on a slide but also for its morphological characteristics, including tumor cell density, structural organization, nuclear texture and chromatin packaging (Figure 4). From this seemingly straightforward observation follows an important implication: even experienced pathologists often find it challenging to reliably identify and interpret subtle morphological features within heterogeneous tumor tissue and to translate them into prognostic predictions. Artificial intelligence may facilitate the identification of subtle morphological patterns and reduce interobserver variability, potentially improving the reproducibility of histopathological assessment [40].

Table 4 summarizes some of the histological features extracted and analyzed by computational models, alongside findings currently assessed in standard human-centered pathology according to CAP protocols [14]. It should be noted that the identified approaches to applying computer vision can be broadly divided into three categories. The first involves a straightforward enhancement of existing morphometric scales. For example, rather than simply assessing tumor size, models quantify tumor area and its proportion across tiles. The second approach also relies on morphometric analysis, but focuses on parameters that are traditionally assessed qualitatively or not evaluated at all. In this way, visual assessment of nuclear morphology is transformed into quantitative metrics such as nuclear density, size, shape, intensity, texture, etc. [15,16]. The same applies to IHC features: whereas modern protocols typically assess the presence of marker expression, computer vision enables the calculation of quantitative biomarkers such as area ratio, proportion and purity [19]. Applying computer vision to IHC slides also opens new perspectives for enabling automated distinction between membranous and cytoplasmic marker expression, which is currently a challenge for pathologists. Overall, this approach not only transforms key morphological descriptors from qualitative observations into quantitative variables, but also enables analysis of previously underexplored features, including spatial relationships between histological classes and metrics related to the stromal component of the tumor. Finally, the third approach, utilized to varying degrees in several studies [6,27,28], involves deep learning-based pattern extraction, in which models are allowed to independently identify morphological features, that may be difficult to recognize visually, but are critical for predicting tumor aggressiveness and patient prognosis. Unlike the first two approaches, this strategy does not rely on pre-existing evaluation and provides opportunities to further explore the patterns that computational models identify when provided with greater autonomy. Overall, these three approaches largely achieve their primary goal of predicting pCR and, in studies comparing computational and human assessments, frequently demonstrate promising predictive performance comparable to or exceeding human assessment in selected tasks. Collectively, these advances suggest that computer vision has the potential not only to refine existing frameworks for histological evaluation, but also to establish new standards for morphological analysis in breast cancer.

A significant finding is that the tumor microenvironment, much like tumor tissue itself, contains valuable predictive markers of therapy sensitivity and resistance. Previous studies have already highlighted the tumor–stroma ratio as a potential marker of survival in breast cancer patients [41]. However, the stromal component remains an underexplored yet promising prognostic feature. Its systematic investigation may contribute to a more comprehensive understanding of tumor morphology and improve predictive modeling in future research.

For practicing pathologists, one of the key areas of interest is the automated analysis of lymphocytes—an important and visually distinctive predictor of therapy sensitivity [42]. This review emphasized not only the prognostic value of TILs but also the advantages of using artificial intelligence for their assessment: several studies have demonstrated, computer-based TIL quantification has demonstrated the potential to improve reproducibility and achieve predictive performance comparable to expert pathological assessment, for whom accurate lymphocyte scoring is often challenging [43]. Moreover, lymphocytes are a heterogeneous cell population, and their subtypes may possibly have distinct and even opposing effects on patient prognosis [44]. The reviewed studies underline the importance of evaluating not only lymphocyte counts but also their localization, antigenic composition and spatial relationships with other histological classes.

An important observation highlighted by several reviewed studies [6,19] is the substantially lower predictive performance reported for TNBC compared to HER2-positive tumors (Table S1). This issue is clinically significant because patients with TNBC, characterized by aggressiveness and historically unfavorable prognosis, derive substantial benefit from NAT [45]. Lower predictive accuracy in TNBC may partially reflect the pronounced intratumoral and microenvironmental heterogeneity of this subtype. Spatial heterogeneity may additionally contribute to sampling bias, as diagnostically relevant regions may be absent from limited biopsy material or small image tiles. Several studies [46,47] investigated additional biomarkers potentially associated with treatment response in TNBC, including Ki-67 expression, androgen receptor status, p53 expression, tumor-infiltrating lymphocytes, particularly CD8+ cells and genomic tumor characteristics. Therefore, one possible solution may involve broadening data modality for TNBC patients. It also must be mentioned that another study discussed in this review [23] that was conducted on TNBC-only cohort of patients showed notably better results (AUC = 0.832 in external validation group). This was achieved by incorporating graphs and considering spatial relations between classes for pCR prediction. Future studies may also benefit from incorporating spatially informed architectures capable of capturing tumor-stroma interactions, larger contextual regions, multi-region sampling strategies.

Another important consideration is the potential clinical value of accurate and reproducible pCR prediction. Beyond prognostic stratification, computational pathology may have direct implications for surgical decision-making following neoadjuvant therapy. Accurate prediction of residual disease and pathological complete response could help identify patients eligible for breast-conserving surgery instead of mastectomy, particularly among patients with locally advanced tumors. The study by Tinterri C., Barbieri E. [48] demonstrated that breast-conserving surgery after neoadjuvant therapy can be oncologically safe in selected cT3–4 patients and is strongly associated with treatment response and ypT0 status. Consequently, reliable artificial intelligence-based prediction models may contribute to treatment de-escalation, optimization of surgical planning and more personalized therapeutic strategies.

The pursuit of a universal approach to tumor biopsy assessment and the development of computer vision technologies capable of overcoming existing limitations and enabling accurate and rapid prediction of pCR remain important and promising directions of research at the intersection of oncology, pathology and computational science. Various computer vision architectures and training strategies have been investigated in attempts to develop models best suited for predicting therapeutic response [49,50], as well as studies on deep learning in histopathology [51], most notable of which are CNN-based architectures and MIL-based models. For a certain period of time, CNNs represented the dominant paradigm in digital pathology due to their ability to efficiently extract local morphological features from histological images. These models demonstrated promising performance in tasks such as tissue segmentation, cell classification and feature extraction. However, CNN-based approaches often require extensive annotation and may have limited ability to capture broader spatial relationships and global tissue context. MIL-based models, on the other hand, have been attracting increasing attention because they enable analysis of whole-slide images without manual annotation and allow integration of information from multiple image regions simultaneously [52]. Such architectures may facilitate identification of subtle morphological patterns associated with treatment response that are difficult to recognize visually. The increased complexity of these models is frequently accompanied by reduced interpretability, which may be one of the principal barriers to their translational potential. However, recent MIL frameworks increasingly incorporate attention mechanisms to enhance both performance and interpretability, therefore these limitation may be overcome in the foreseeable future.

The current state of the field is increasingly defined by hybrid architectures that aim to combine the complementary strengths of different paradigms. In particular, transformer-based models are being integrated into MIL pipelines to capture long-range dependencies and CNN-transformer hybrids are being developed to balance local feature extraction with global context awareness [52,53]. This trend suggests that the integration of multiple model architectures may be key to capturing diverse biological signals while preserving sufficient interpretability for clinical practice. Nevertheless, the field continues to evolve rapidly toward increasingly integrative and biologically informed modeling strategies.

Several of the studies discussed in this review demonstrated the added value of incorporating not only WSI, but also IHC, genomic and radiological data. A number of works in the past similarly applied machine learning for prognosis prediction using radiological data, particularly MRI, both in breast cancer [54] and in other malignancies [55,56]. Some MRI-based models have achieved very high predictive accuracy, with AUC values above 0.9 [57], and an exceptionally high predictive performance (AUC = 0.978) was obtained using transcriptomic data [58]. Notably, this work considered not only the mutational and metabolic profiles of tumor cells but also the cellular composition of the tumor microenvironment. Other publications have trained models exclusively on clinical and pathological data and also reported promising results [59,60]. Taken together, these findings suggest that a cross-modal approach may substantially improve the accuracy of therapy response prediction, enable the development of more personalized and effective treatment plans and advance broader understanding of breast cancer biology.

Many of the reviewed studies reported encouraging results. However, current evidence on the application of computational pathology for NAT response prediction in breast cancer remains highly heterogeneous and should be interpreted with caution. The included studies differed substantially in cohort size and composition, molecular subtype distribution, staining protocols, endpoint definitions, image preprocessing pipelines and validation strategies, limiting direct comparison between models (see Table S1). Several studies relied exclusively on internal cross-validation or relatively small held-out test sets, whereas truly independent external validation was performed less frequently. In addition, many cohorts were relatively small, increasing the risk of overfitting and limiting the generalizability of the reported predictive performance.

Several studies aimed at direct standardized comparisons between artificial intelligence models and pathologists and demonstrated that computational models may achieve promising results in tasks such as TIL quantification and pCR prediction. However, pathologists continue to serve as the reference standard for annotation, diagnosis, region selection and interpretation of histopathological findings. In a number of studies, informative regions or tiles were manually selected by pathologists prior to model training, potentially introducing selection bias and partially reflecting the perspective of individual experts. Furthermore, many deep learning models remain insufficiently interpretable, with biologically meaningful morphological patterns often identified only retrospectively through feature analysis or heatmap visualization. Therefore, computational pathology should currently be regarded primarily as a complementary tool capable of improving reproducibility, standardization and efficiency of pathological assessment rather than a competition to expert human evaluation.

## 6. Conclusions

In conclusion, the design of predictive studies and models intended for clinical application must consider multiple layers of information: not only the structure of tumor tissue and individual tumor cells, but also the tumor microenvironment; not only the quantitative representation of histological classes, but also their spatial relationships; not only the presence of inflammatory infiltration, but also the localization and cellular composition of immune infiltrates. For this reason, progress in the field of computational pathology will depend on multimodal strategies and the development of specialized parallel models capable of integrating diverse biological signals.

## Figures and Tables

**Table 4:** Comparison of AI-extracted histological features and conventional CAP-based findings used to assess tumor aggressiveness and predict response to neoadjuvant therapy in breast cancer.

AI-Extracted Histological Features	CAP-Assessed Histological Findings
Composite deep features reflecting cellular atypia, tissue organization and architectural patterns (solid, cord-like, trabecular structures)	Tubule/glandular formation
Tumor area and proportion across tiles	Tumor size (in biopsy context: extent of involvement)
Nuclear density, orderliness, spatial arrangement, size, shape, intensity, texture, heterogeneity	Nuclear morphology and pleomorphism
Quantitative tumor cell density per tile	Tumor cellularity assessment
Necrosis proportion on pixel or tile level	Presence of necrosis
Automated lymphocyte detection and count; spatial distribution of lymphocytes; lymphocyte–tumor proximity metrics	Tumor-infiltrating lymphocytes assessment, their localization
Quantitative IHC features (area ratio, proportion, purity)	IHC marker expression (Ki-67, ER, HER2, PD-L1)
Tumor–stroma ratio; stromal texture; stroma classification based on predominant component (collagen, fibroblast or lymphocyte)	Not routinely assessed

## Data Availability

No new data were created or analyzed in this study.

## Supplementary Materials

The following are available online at https://www.mdpi.com/article/10.3390/cancers18111857/s1, Table S1: Summary of cohort characteristics, validation strategies, and major methodological limitations of the studies included in this review.

## Abbreviations

The following abbreviations are used in this manuscript:

NAT	Neoadjuvant therapy
TNBC	Triple-negative breast cancer
pCR	Pathological complete response
HER2	Human epidermal growth factor receptor 2
ER	Estrogen receptor
WSI	Whole-slide image
ROI	Region of interest
AUC	Area under the curve
CNN	Convolutional neural network
IHC	Immunohistochemistry
H&E	Hematoxylin and eosin
CLAM	Clustering-constrained-attention multiple-instance learning
SVM	Support vector machines
RF	Random forest
TILs	Tumor-infiltrating lymphocytes
MRI	Magnetic resonance imaging
AI	Artificial intelligence
CAP	College of American Pathologists
RCB	Residual cancer burden
MIL	Multi-instance learning
