# Supplementary material for: Computer Vision for Predicting the Efficacy of Neoadjuvant Therapy in Breast Cancer
Source: Cancers (Basel). 2026 Jun 5;18(11):1857. doi: 10.3390/cancers18111857 (PMC13256785; doi:10.3390/cancers18111857)
Supplement: Supplementary file 1 [file cancers-18-01857-s001.zip › cancers-4295923-supplementary.pdf]

References	Sample size	Validation	Main limitations
[6]	Training: 438 (PRIMUNEO, 3-fold CV); Test: 490 (CGFL, external)	Internal: 3-fold CV; external: single-center CGFL (different hospital)	Preprint study; potential bias from varied chemotherapy regimens
[15]	Total: 58; No train/test split	Internal: resampling/bootstrap; no external	Small sample size; no external validation; only HER2+ and TNBC; only five predefined nuclear features
[16]	Training: 111; Test: 38	Internal: 5-fold CV + independent test set; no external	No external validation; small sample size
[17]	Training: 432; Test: 107	Internal: separate validation dataset (107, 80/20 split) + 16× repeated subsampling; no external	No external validation; requires data analyst expert
[18]	Training: 352; Test: 539; Additional external: 126 (IMPRESS)	Internal: 539 patients (three centers); external: IMPRESS (different scanners, subtypes)	Misses single lymphocytes; predictive performance varies across cohorts/centers; tumor amount influences prediction; external dataset uses different scanners and subtypes (HER2+ not in training)
[19]	Training and test (study cohort): 126 (LOOCV); External: 40	Internal: LOOCV; external: two independent cohorts (20 HER2+, 20 TNBC, balanced)	Moderate cohort size; poor TNBC external validation (AUC = 0.59)
[20]	Training: 57; Test: 19	Internal: 3-fold CV; no external	Preprint study; small sample size; TNBC only; no external validation; may ignore patch-level heterogeneity
[21]	Training: 165; Test: 78	Internal: 10-fold CV; no external	Small test cohort; no external validation; TNBC only
[22]	Training: 535; Test: 118; External: 907; Prospective: 110	Internal: 10-fold CV + internal test (118); external: 6 Chinese centers; prospective: 110 consecutive	Treatment regimens not fully standardized per patient
[23]	Training: 85; Test: 79 (external)	Internal: 8-fold CV (tile) + LOO-CV (patient); external: Galway (Ireland)	Small sample size; slide quality and staining variability; class imbalance among 16 histology types; TNBC only

[24]	Training: 482;<br>Test: 392 (VC1-3)	Internal: 10-fold CV; external: three cohorts (VC1-3)	Patient heterogeneity across centers
[25]	Training: 207;<br>Test: 103	Internal: train/test split (70/30); no external	No external validation; SVM/RF require manual annotation; moderate test set
[26]	Training: 391 (unifocal, augmented);<br>Test: 51 (TC1 unifocal), 105 (TC2 multifocal)	Internal: 49 validation (unifocal) + two internal test sets (same center); no external	No external validation; manual ROI selection; not all NAT regimens included; no analysis by molecular subtype
[27]	Training: 261;<br>Test: 107 (VC1), 72 (VC2)	External: two independent centers (VC1, VC2); no internal validation set	Manual ROI selection; uneven molecular subtype distribution across cohorts
[28]	Training: 140;<br>Test: 61	Internal: random split (70/30); no external	No external validation; imbalance of molecular subtypes between training and test sets; manual ROI selection
[31]	Training: 695;<br>Test: 340 (V1-3)	Internal: 5-fold CV; external: three centers (V1-3)	Manual ROI annotation; potential selection bias
[32]	Total: 120;<br>Training/test numbers not fixed	Internal: random splits (60:40, 80:20); no external	No external validation; moderate sample size; manual patch annotation; TNBC only; missing data reduced cohort to 98 patients
[37]	Total: 113 pre-treatment samples (SWOG S0800);<br>No train/test split	Internal: ROC analysis on same cohort; no external	No external validation; artifacts can cause false positives; only HER2-negative patients; moderate sample size
[38]	DL performance: 210 concordant WSIs; therapy prediction: 203	Internal: CCC on 210 concordant cases; no external for DL model	No external validation; small sample size for TNBC (n=55); performance poorer in high sTIL cases; manual quality control required
[39]	Total: 623<br>pre-treatment biopsies	Internal: logistic regression on whole cohort; no CV or test set	No external validation; substantial loss of observations in multivariate analysis (1/3)

Supplementary Table S1. Summary of cohort characteristics, validation strategies, and major methodological limitations of the studies included in this review.

Abbreviations: CV — cross-validation; LOOCV — leave-one-out cross-validation; CCC — concordance correlation coefficient; ROI — region of interest; RF — random forest; SVM — support vector machine.
